# Neck circumference as an independent predictive contributor to cardio-metabolic syndrome

**DOI:** 10.1186/1475-2840-12-76

**Published:** 2013-05-16

**Authors:** Jing-ya Zhou, Hui Ge, Ming-fan Zhu, Li-jun Wang, Li Chen, Yao-zong Tan, Yu-ming Chen, Hui-lian Zhu

**Affiliations:** 1 Guangdong Provincial Key Laboratory of Food, Nutrition and Health, School of Public Health, Sun Yat-Sen University, 74th Zhongshan Road II, Guangzhou 510080, People’s Republic of China; 2 Health Examination Centre, First Affiliated Hospital of Sun Yat-sen University, 58th Zhongshan Road II, Guangzhou 510080, People’s Republic of China

**Keywords:** Neck circumference, Cardio-metabolic risk factors, Central obesity

## Abstract

**Background:**

The predictive potentials of neck circumference (NC) for cardio-metabolic risks remain uncertain. The aim of this study was to investigate whether NC independently contributes to the prediction of cardio-metabolic risks beyond body mass index (BMI), waist circumference (WC) and waist to hip ratio (WHpR) in a large Chinese population.

**Methods:**

A total of 4201 participants (2508 men and 1693 women) aged 20-85 were recruited from the Health Examination Centre between May 2009 and April 2010, anthropometric indices, biochemical and clinical parameters were measured. Receiver operating characteristic, partial correlation and logistic regression analyses were employed to evaluate the association of the anthropometric indices to cardio-metabolic risks separately by gender.

**Results:**

Neck circumference was positively correlated with SBP and DBP (r=0.250 and 0.261), fasting blood glucose (FBP) (r=0.177), TG (r=0.240), TC (r=0.143) and LDL-C (r=0.088) and negatively correlated with HDL-C (r=-0.202) in males (all P<0.01). Similar results were found in females with the exception of TC. The AUCs of NC for metabolic abnormalities ranged from 0.558 (Increased LDL-C) to 0.683 (MS-rf) in men and 0.596 (Increased LDL-C) to 0.703 (MS-rf) in women (P<0.01). The NC of ≥37 cm for men and ≥33 cm for women were the best cut-off points for metabolic syndrome. The adjusted ORs (95% CIs) of NC in men and women respectively were 1.29 (1.12-1.48) and 1.44 (1.20-1.72) for metabolic syndrome risk factors (MS-rf), 1.15 (1.01-1.32) and 1.22 (1.03-1.46) for high BP, 1.16 (1.02-1.33) and 1.42 (1.18-1.71) for increased TG, and 1.26 (1.06-1.50) and 1.32 (1.06-1.65) for increased FBP; the adjusted OR of NC in women for decreased HDL-C was 1.29 (1.10-1.51).

**Conclusions:**

Neck circumference was significantly associated with cardio-metabolic risk factors and independently contributed to the prediction of cardio-metabolic risks beyond the classical anthropometric indices in adults of China.

## Background

Cardio-metabolic syndrome is a cluster of metabolic abnormalities including glucolipid metabolism disorders, overweight, insulin resistance and hypertension [1]. Obesity, especially central obesity, is the predominant determinant of metabolic syndrome (MS) [2-4]. Many simple anthropometric indices, including the body mass index (BMI), waist circumference (WC), waist-to-hip ratio (WHpR) and waist-to-height ratio (WHtR), are widely used as markers to reflect obesity or central obesity and predict MS or other cardiovascular risks [5-7]. Recently, neck circumference (NC), as a simple and time-saving anthropometric measurement, was identified as an index of central obesity and a promising potential predictor for cardio-metabolic syndrome [5,8]. However, whether its predictive potential for cardio-metabolic risks is comparable or superior to the above-mentioned indices and whether NC has an independent contribution to the prediction beyond the classic anthropometric indices are still unclear.

Growing evidences from specific populations have supported the hypothesis that the larger the neck circumference, the higher the cardio-metabolic risk. Selim et al. [9] revealed that NC was positively correlated with the occurrence of metabolic abnormalities in obese children. In another study on 1912 middle-aged and elderly Turkish adults [10], NC had a greater value than WC regarding the association with MS. Moreover, the results from the Framingham Heart Study [11] further demonstrated that NC was independently associated with risk factors of cardiovascular disease beyond visceral adipose and BMI. Up to now, the clinical significance of NC regarding to cardio-metabolic syndrome in population-based studies is extremely limited and needs to be further clarified. Since the predictive potential of classic anthropometric indices (e.g., BMI, WC) for cardio-metabolic risks varied among different ethnic populations [12,13], it was still speculative that whether the NC-MS association could be generalized to other ethnic populations.

The present study aims to investigate whether NC independently contributes to the prediction of the risk of cardio-metabolic syndrome in a large Chinese population. This study will present a novel approach for screening cardio-metabolic risks.

## Methods

### Study population and basic information

A total of 4771 participants in this cross-sectional study were enrolled from the Health Examination Centre of the First Affiliated Hospital of Sun Yat-sen University between May 2009 and April 2010. In addition to completing a medical history survey and physical examination, including body height, body weight, NC, WC and hip circumference (HC), all subjects also underwent an overnight fasting blood draw for the determination of biochemical indicators. We excluded those subjects with a history of malignancy and thyroid diseases (n=36) or subjects taking medicine for high blood pressure, diabetes, dyslipidemia (n=486) or weight control (n=48). Finally, 4201 subjects (2508 men and 1693 women, aged 20 years or older) were included in our analysis. All participants signed an informed consent form, and the study protocol was approved by the ethics committee of the First Affiliated Hospital of Sun Yat-sen University.

### Measurements

The physical examination was performed with the subjects after an overnight fast and standing upright with their shoulders relaxed by one investigator using standard techniques as follows. Weight was measured using a digital scale (Hanson, Watford, Hertforshire, England) to within 0.1 kg with only undergarments, and height was determined using a portable stadiometer (Holtain, Crymmych, Wales) to within 0.1 cm in bare feet. Waist and hip circumferences were measured at the midpoint between the lowest rib and the iliac crest and at the greater trochanter, respectively, with plastic tapes calibrated weekly to within 1 mm. Neck circumference was measured as indicated in the previous report [5], to within 0.5 mm using a plastic tape which was also calibrated weekly. Briefly, participants were asked to stand erect with their head positioned in the Frankfort horizontal plane. The superior border of a plastic tape was placed just below the laryngeal prominence and applied perpendicular to the long axis of the neck. The BMI (weight divided by the square of height, kilograms per square meter [kg/m^2^]) and WHpR (WC divided by HC) values were calculated. BP was measured at the subjects’ right hand with the subjects sitting after 5 min of rest (15 to 30 min were needed for some subjects who habitually smoked or performed strenuous exercise) using a calibrated sphygmomanometer (Hawksley, WA Baum Co, USA). The criteria for systolic blood pressure (SBP) and diastolic blood pressure (DBP) were the first and fifth phase Korotkoff sounds, respectively. All the measurements of anthropometric indices and BP were done by well-trained physicians, nurses or research staff.

Blood samples were collected and sent to the biochemistry laboratory in the hospital for analysis. An enzymatic calorimetric test was used to measure the total cholesterol (TC) and triglyceride (TG) concentrations. The selective inhibition method was used to measure the level of high-density lipoprotein cholesterol (HDL-C), and a homogeneous enzymatic calorimetric test was used to measure the level of low-density lipoprotein cholesterol (LDL-C) (Advia1650 Autoanalyzer, Byer Diagnostics Leverkusen Germany). Apolipoprotein A, Apolipoprotein B and Apolipoprotein E were determined by immunoturbidimetric assays.

### Definition of cardio-metabolic risk factors

The definition of MS we adopted in this study was promulgated by the International Diabetes Federation (IDF) — the IDF criteria. According to the IDF definition of MS, to confirm a person who has MS, he must be diagnosed with central obesity based on WC (WC ≥ 90 cm for men; WC ≥ 80 cm for women in a Chinese population), with the addition of at least two of the following four factors: increased TG (≥ 1.7 mmol/L), decreased HDL-C (≤ 1.03 mmol/L for men and ≤ 1.29 mmol/L for women), high BP (SBP ≥ 130 or DBP ≥ 85 mmHg) and increased FBP (≥ 5.60 mmol/L) [1]. All four factors were required to have been detected for the first time during the evaluation for this study or have been previously detected but not controlled with drugs.

The aim of this study was to investigate the independent contribution of NC to cardio-metabolic risk factors, and WC is a condition necessary to diagnose metabolic syndrome according to the IDF criteria. Therefore, when we compared the correlation of NC, BMI, WC and WHpR to cardio-metabolic risks, we did not include WC as the risk index of cardio-metabolic risk in this study. So, metabolic syndrome risk factors (MS-rf) employed in this study were defined with an index of ≥ 2 risk factors for MS based on the IDF criteria except for central obesity.

According to the China Adult Dyslipidemia Prevention Guide [14], increased TC was defined as TC ≥ 5.18 mmol/L, and increased LDL-C was defined as LDL-C ≥ 3.37 mmol/L.

### Statistical analysis

All data were input using the EPI-data 3.0 software-specified unified input interface by two statisticians and analyzed using SPSS16.0 statistical software (SPSS Inc., Chicago, IL, USA). All data were analyzed and reported by sex. The means and standard deviations were used to describe continuous data. For categorical data, frequencies and percentages were estimated. The associations between cardio-metabolic risk factors and anthropometric indices were assessed using partial correlation analysis. Receiver operating characteristic (ROC) analyses were performed to assess the accuracy of the anthropometric indices as diagnostic tests for cardio-metabolic syndrome and determine optimal sex-specific NC cut-offs in relation to MS. Odds ratios (ORs) and 95% confidence intervals (CIs) were obtained by univariate and multivariate logistic regression analyses in models that were unadjusted or adjusted for age and other anthropometric confounders. All tests of significance were two tailed with a value of p<0.05.

## Results

The anthropometric and metabolic characteristics of the participants by gender are presented in Table 1. The 4201 subjects had a mean age of 43.7±12.0 years (men: 44.1±12.4, women: 43.1±11.3 years), mean BMI of 23.73±3.15 kg/m^2^ (men: 24.44±2.98, women: 22.67±3.10 kg/m^2^) and mean WC and NC of 82.37±10.08 cm (men: 86.44±8.71, women: 76.36±8.88 cm) and 35.41±3.39 (men: 37.40±2.46, women: 32.46±2.24 cm), respectively. Of the 4201 subjects, MS was diagnosed in 443 men (17.66%) and 215 women (12.70%). High BP was diagnosed in 965 men (38.48%) and 429 women (25.34%). Increased FBG was found in 349 (13.92%) men and 150 (8.86%) women. Increased TG was found in 972 men (38.76%) and 287 women (16.95%). Decreased HDL-C was found in 646 men (25.76%) and 471 women (27.82%). Increased LDL-C was detected in 1395 men (55.62%) and 743 women (43.89%). Increased TC was detected in 1633 men (65.11%) and 1044 women (61.79%).

**Table 1:** Characteristics of the study participants (n=4201)

	**Male (n=2508)**		**Female (n=1693)**	
	**Mean**	**SD**	**Mean**	**SD**
Continuous data				
Age (year)	44.1	12.4	43.1	11.3
Height, cm	169.4	5.8	158.1	5.4
Weight, kg	70.23	9.85	56.69	8.20
Neck circumference, cm	37.40	2.46	32.46	2.24
Waist circumference, cm	86.44	8.71	76.36	8.88
Hip circumference, cm	96.50	5.87	92.85	5.99
Body mass index, kg/m^2^	24.44	2.98	22.67	3.10
Waist-to-hip ratio	0.89	0.06	0.82	0.06
SBP, mmHg	125.0	16.1	118.8	18.5
DBP, mmHg	78.3	11.6	72.8	10.7
TG, mmol/L	1.81	1.36	1.24	0.93
TC, mmol/L	5.59	1.09	5.51	1.08
HDL-C, mmol/L	1.25	0.36	1.52	0.38
LDL-C, mmol/L	3.51	0.97	3.33	0.93
Glucose, mmol/L	5.08	1.22	4.90	0.77
ApoA, g/L	1.19	0.32	1.37	0.39
ApoB, g/L	0.96	0.28	0.89	0.23
ApoE, g/L	43.53	14.59	43.10	12.85
Categorical data	n	%	n	%
MS	443	17.66	215	12.70
High BP	965	38.48	429	25.34
Increased FBG	349	13.92	150	8.86
Increased TG	972	38.76	287	16.95
Decreased HDL-C	646	25.76	471	27.82
Increased LDL-C	1395	55.62	743	43.89
Increased TC	1633	65.11	1046	61.78

Abbreviations: *SD*, standard deviation.

The partial correlations between cardio-metabolic risk factors and anthropometry indices by gender are shown in Table 2. In male subjects, after adjusting for age, the NC revealed a positive correlation with SBP and DBP (r=0.250 and 0.261, both P<0.01), FBG (r=0.177, P<0.01), TG (r=0.240, p<0.01), TC (r=0.143, p<0.01) and LDL-C (r=0.088, p<0.01). In contrast, NC was negatively correlated with HDL-C (r=-0.202, p<0.01). Similarly, in female subjects, NC showed the same trend in the correlation with cardio-metabolic risk factors, with the exception of TC. The correlations of other anthropometric indices to the cardio-metabolic risk factors were the same as those of NC to the cardio-metabolic risk factors in both genders.

**Table 2:** Partial correlation coefficients between simple anthropometric indices and cardio-metabolic risk factors (adjusted for age) (n=4201)

	**NC**	**WC**	**BMI**	**WHpR**	**HC**	**WT**
Male (n=2508)						
SBP	0.250^b^	0.257^b^	0.303^b^	0.193^b^	0.235^b^	0.259^b^
DBP	0.261^b^	0.276^b^	0.303^b^	0.225^b^	0.230^b^	0.280^b^
FBG	0.177^b^	0.185^b^	0.142^b^	0.208^b^	0.099^b^	0.133^b^
TG	0.240^b^	0.281^b^	0.251^b^	0.282^b^	0.187^b^	0.226^b^
HDL-C	−0.202^b^	−0.271^b^	−0.232^b^	−0.258^b^	−0.196^b^	−0.218^b^
TC	0.143^b^	0.170^b^	0.160^b^	0.171^b^	0.113^b^	0.129^b^
LDL-C	0.088^b^	0.133^b^	0.121^b^	0.121^b^	0.103^b^	0.109^b^
Female (n=1693)						
SBP	0.255^b^	0.239^b^	0.232^b^	0.177^b^	0.199^b^	0.197^b^
DBP	0.189^b^	0.194^b^	0.196^b^	0.150^b^	0.157^b^	0.180^b^
FBG	0.180^b^	0.218^b^	0.206^b^	0.179^b^	0.159^b^	0.206^b^
TG	0.199^b^	0.200^b^	0.176^b^	0.191^b^	0.124^b^	0.174^b^
HDL-C	−0.234^b^	−0.269^b^	−0.217^b^	−0.261^b^	−0.169^b^	−0.219^b^
TC	0.039	0.034	0.084^b^	0.039	0.015	0.037
LDL-C	0.075^b^	0.082^b^	0.128^b^	0.064^a^	0.070^a^	0.081^b^

a: p<0.05, b: p<0.01.

The areas under the ROC curves (AUCs) were constructed to evaluate the predictive values of anthropometric indices for cardio-metabolic risks (Table 3). The AUCs of NC were 0.635 and 0.659 for the risk factor of BP, 0.659 and 0.678 for TG, 0.618 and 0.641 for HDL-C, 0.624 and 0.646 for glucose, 0.558 and 0.596 for LDL-C and 0.683 and 0.703 for MS-rf in men and women, respectively. An NC of ≥37 cm for men and ≥33 cm for women were the best values of combined sensitivity and specificity in identifying MS. The AUCs of NC for all cardio-metabolic risk factors, except BMI for glucose in men and BMI for HDL-C in women, were lower than those of other anthropometric measurements, including WC, BMI and WHpR.

**Table 3:** Areas under the ROC by anthropometric indices for cardio-metabolic risk factors (n=4201)

	**NC**	**WC**	**BMI**	**WHpR**
	**AUC (95% CI)**	**AUC (95% CI)**	**AUC (95% CI)**	**AUC (95% CI)**
Male (n=2508)				
MS-rf	0.683 (0.662-0.705)	0.715 (0.695-0.736)	0.699 (0.678-0.720)	0.716 (0.696-0.736)
High BP	0.635 (0.613-0.657)	0.652 (0.630-0.674)	0.649 (0.627-0.671)	0.650 (0.628-0.672)
Increased TG	0.659 (0.637-0.681)	0.693 (0.672-0.699)	0.678 (0.657-0.699)	0.689 (0.669-0.710)
Decreased HDL-C	0.618 (0.593-0.643)	0.640 (0.616-0.664)	0.631 (0.607-0.655)	0.638 (0.614-0.662)
Increased FBG	0.624 (0.592-0.656)	0.661 (0.631-0.691)	0.617 (0.586-0.649)	0.703 (0.674-0.732)
Increased LDL-C	0.558 (0.536-0.581)	0.580 (0.557-0.602)	0.574 (0.552-0.597)	0.578 (0.555-0.600)
Female (n=1693)				
MS-rf	0.703 (0.672-0.735)	0.764 (0.738-0.791)	0.723 (0.693-0.752)	0.766 (0.740-0.792)
High BP	0.659 (0.629-0.689)	0.733 (0.706-0.760)	0.699 (0.670-0.728)	0.731 (0.705-0.758)
Increased TG	0.678 (0.645-0.712)	0.734 (0.704-0.763)	0.691 (0.659-0.723)	0.738 (0.709-0.768)
Decreased HDL-C	0.641 (0.612-0.670)	0.665 (0.637-0.692)	0.640 (0.612-0.669)	0.667 (0.639-0.695)
Increased FBG	0.646 (0.595-0.697)	0.715 (0.672-0.758)	0.670 (0.624-0.716)	0.706 (0.662-0.750)
Increased LDL-C	0.596 (0.569-0.623)	0.651 (0.625-0.677)	0.638 (0.612-0.665)	0.641 (0.615-0.667)

Abbreviations: *ROC*, receiver operating characteristic; *AUC*, area under the curve; *CI*, confidence interval.

**Figure 1:**
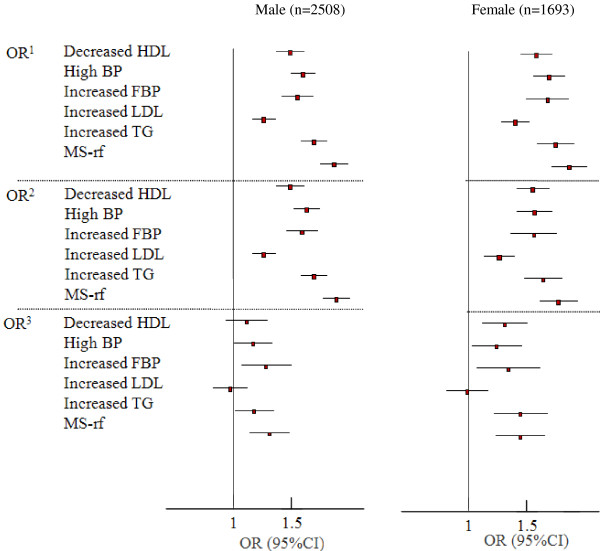
**Gender-specific odds ratios (ORs) and 95% confidence intervals (95% CIs) of cardio-metabolic risk factors for neck circumference (NC).** OR^1^: unadjusted, OR^2^: adjusted for age, OR^3^: adjusted for age, BMI, WC and WHpR.

The crude and adjusted ORs for the cardio-metabolic risk factors for per SD increase in NC are presented in Figure 1. Generally, greater NC was linearly associated with higher risk of metabolic abnormalities in both genders. Women tended to have closer associations than men. Based on the univariate logistic regression analysis with MS-rf as the dependent variable, the crude OR was 2.03 (95% CI: 1.84-2.23) in men and 2.03 (95% CI: 1.79-2.29) in women (p<0.01 in both).

After adjusting for age, NC was significantly associated with MS-rf at ORs 2.06 in men and 1.88 in women (P<0.01 in both). With further adjustments for BMI, WC and WHpR, the significant associations remained between NC and MS-rf with ORs of 1.29 in men and 1.44 in women (P<0.01 in both). In the multivariate model including age, BMI, WC and WHpR, NC also showed obvious correlations with the occurrence of high BP (OR, 1.15 in men and 1.22 in women), increased TG (OR, 1.16 in men and 1.42 in women), increased FBP (OR, 1.26 in men and 1.32 in women) and decreased HDL-C (OR, 1.29 in women). We also made the adjustments for anthropometric indices (BMI, WC and WHpR) one by one due to close colinearity among them, similar results were found to those observed in the previous multivariate model included all of them (data not shown).

## Discussion

The aim of the present study was to determine whether neck circumference (NC) was associated with cardio-metabolic risks and contributed to the prediction beyond the classical anthropometric indices in a cross-sectional survey of 4201 individuals from China. We found that NC was significantly associated with cardio-metabolic risk factors and independently contributed to predicting the likelihood of cardio-metabolic risks both in males and females.

### Neck circumference and cardio-metabolic risks

Based on previous studies, obesity is widely accepted to be associated with metabolic disorders and cardiovascular risk factors [15]. Although BMI, WC and WHpR are widely used anthropometric indices to reflect obesity and predict cardio-metabolic risks [12,16,17], an increasing number of studies have suggested that NC is a simpler, more innovative and practical anthropometric parameter. WC measurements are easily affected by being full or hungry, respiratory movement and wearying heavy clothing, whereas these problems can be avoided in the NC measurement. Therefore, NC was a more reliable anthropometric indice to indicate central obesity [18]. In the Framingham Heart Study, including 2732 subjects (mean age: 57 y), Preis et al [11,19] found that NC was positively associated with risks of type 2 diabetes, hypertension, decreased HDL-C and increased TG. After further adjustments for BMI and WC, NC remained associated with type 2 diabetes. Similar results were observed in a Turkish Adult Cohort Study in 1912 middle-aged and elderly individuals [10]. In our study, we found that NC was significantly correlated with all outcomes of cardio-metabolic risks in both genders, which was in line with the previous studies [20,21]. NC might reflect fat deposition at an ectopic site such as observed in fatty liver [22]. Therefore, the thicker the NC, the greater the risk of cardio-metabolic syndrome is.

### Comparison of associations among the anthropometric indices

Next, we calculated AUCs to evaluate the predictive values of NC and other anthropometric indices for cardio-metabolic risks. According to the ROC analyses between anthropometric indices and MS-rf, the NC presented a significantly large AUC (men: 0.683, women: 0.703), but the values were relatively lower than those of WHpR (0.716 and 0.766), WC (0.715 and 0.764) and BMI (0.699 and 0.723). Up to date, few studies have compared the effect size of NC with WC, BMI, WHtR or other anthropometric indices. Some studies [11,20,21], but not all [10,23], observed a less strong association in NC than those of BMI [11], WHpR [20] and WC [21] with metabolic abnormalities. From these results, we could not consider NC superior to WC, BMI and WHpR to predict cardio-metabolic risks.

It is well established that visceral fat (or central obesity) has greater effect on the development of metabolic abnormalities than subcutaneous fat (general obesity) [24]. WC, WHpR, sagittal abdominal diameter and visceral adiposity index are widely used markers of visceral fat [13,25-27]. These indices are more closely correlated to visceral fat than BMI and NC [13,21]. A variety of studies have demonstrated that these indices of visceral fat have stronger association with metabolic abnormalities or CVD risks than BMI [13,28]. Therefore, it was likely that the inefficient predictive values of NC (vs. WC or WHtR) for MS in this study were due to its poor correlation with visceral fat.

### Optimal cut-off points and independent contribution of neck circumference

We observed optimal NC cut-offs of ≥37 cm in men and ≥33 cm in women for the prediction of MS in this population. Greater values were found in men than in women, which was in consistent with previous study [29]. However, much greater NC cut-offs in both gender were observed in Brazil study (>40 cm in men and > 36 cm in women) for the prediction of MS and insulin resistance [29]. Larger cut-off values of NC for the prediction of MS were also noted in a Turkey population [10]. Greater differences in body size might partially explain the heterogeneity in the optimal cut-offs of NC as WC [30] among different populations. In this regard, ethnic-specific cut-offs of NC would be required for the prediction of cardio-metabolic abnormalities.

Furthermore, the cardio-metabolic risk factors were categorized as dichotomous variables, and their associations with NC were evaluated by logistic regression analysis. The ORs found that the associations between NC and cardio-metabolic risk factors were similar for both genders. Originally, NC was significantly associated with the likelihood of MS-rf and each component of cardio-metabolic syndrome in the model adjusted for age. Subsequently, when controlled for age, BMI, WC and WHpR, the measurement of NC showed significant independence of association regarding the likelihood of cardio-metabolic risk. Thus, we could conclude from these results that NC contributed to cardio-metabolic risk independently of other anthropometric indices in Chinese population, which was in accordance with a previous diabetic population-based study [29]. A measurement of NC might yield additional information in terms of the identification of cardio-metabolic syndrome.

### Limitations

The present study has some limitations. First, this cross-sectional design study limited extension of its interpretation to the causality of associations. Second, all the participants were from the same health examination center, and a selective bias could not be excluded. Finally, because the survey was completed in a single visit, the inherent variability in laboratory tests and measurements could not be taken into account. Despite these limitations, our study has the advantage of introducing a simple and inexpensive method to predict cardio-metabolic risks in a large population. However, because the study was limited to the representativeness of the study sample and cross-sectional study design, further longitudinal studies in representative populations are required to obtain more conclusive results to establish NC as a basic criterion in the diagnosis of cardio-metabolic syndrome.

## Conclusion

Our findings suggest that NC is a simple and effective anthropometric indice to identify cardio-metabolic syndrome among adults of China. NC plays an independent contribution to predicting the metabolic abnormalities beyond the classical anthropometric indices of BMI, WC and WHpR, and may be used as an optimal screening tool for cardio-metabolic risks and other obesity-related chronic diseases.

## Abbreviations

NC: Neck circumference; MS: Metabolic syndrome; MS-rf: Metabolic syndrome risk factors; WC: Waist circumference; BMI: Body mass index; WHpR: Waist-to-hip ratio; WHtR: Waist-to-height ratio; HC: Hip circumference; WT: Weight; SBP: Systolic blood pressure; DBP: Diastolic blood pressure; FBG: Fasting blood glucose; TG: Triglyceride; TC: Total cholesterol; HDL-C: High-density lipoprotein cholesterol; LDL-C: Low-density lipoprotein cholesterol; ApoA: Apolipoprotein A; ApoB: Apolipoprotein B; ApoE: Apolipoprotein E.

## Competing interests

The authors declare that they have no competing interests.

## Authors’ contribution

Hui-lian Zhu made substantial contributions to conception and design. Hui Ge, Ming-fan Zhu, Li-jun Wang, Li Chen and Yao-zong Tan performed the acquisition of data. Jing-ya Zhou, Hui-lian Zhu and Yu-ming Chen performed the analysis and interpretation of date. Jing-ya Zhou and Hui-lian Zhu were involved in drafting the manuscript. Hui-lian Zhu gave the final approval of the version to be published. All authors read and approved the final manuscript.
